# Development of a Methodology for Assessing Mechanical Damage in Biological Objects: Impact Parameters and Micro-Damage Analysis

**DOI:** 10.3390/ma18092075

**Published:** 2025-05-01

**Authors:** Serhii Kharchenko, Sylwester Samborski, Rafat Al Afif, Farida Kharchenko, Mariusz Kłonica, Mykhailo Piven

**Affiliations:** 1 Department of Fundamentals of Production Engineering, Lublin University of Technology, 20-618 Lublin, Poland; s.samborski@pollub.pl (S.S.); m.klonica@pollub.pl (M.K.); 2 Institute of Chemical and Energy Engineering, University of Natural Resources and Life Sciences, 1190 Vienna, Austria; rafat.alafif@boku.ac.at; 3 Department of Agricultural Engineering, Sumy National Agrarian University, 40-021 Sumy, Ukraine; faridakharchenko@gmail.com; 4 Department of Physics and Mathematics, State Biotechnological University, 61002 Kharkiv, Ukraine; m.v.piven@gmail.com

**Keywords:** biological object, static and dynamic load, tomographic analysis, Monte Carlo method, microdamage coefficient

## Abstract

Mechanical impacts on loose biological objects caused by technological equipment can result in both external and internal damage, compromising the quality, storage life, and reproductive capacity of biological materials. This study addresses the need for a reliable methodology to assess such damage. The research aims to develop a systematic approach for identifying damage parameters in biological objects. The methodology involves applying artificial loading to biological samples, determining destructive forces, conducting tomography, processing images, and evaluating damage extent. Experiments were performed using a standard material testing machine and a custom-built impact test bench with varying parameters such as static and dynamic characteristics, object orientation, and load magnitude. The microstructure of the sample, in the form of 2D cross-sections and 3D images, was obtained using X-ray computed tomography. Image processing, with the Monte Carlo method, allowed for the calculation of microdamage coefficients. The key result of this study is the identification of a relationship between the microdamage coefficient of corn seeds and external load parameters. These findings are critical for understanding the effects of mechanical impact on biological materials. Future research should focus on expanding the study to other biological objects and enhancing measurement techniques for more precise damage assessment.

## 1. Introduction

Productivity and quality of technological processes of loose material processing and minimization of energy consumption determine not only the economic profitability of production facilities but also compliance with the norms and standards of final products for consumers, environmental protection requirements, and human health and safety standards [1,2]. Technological processes such as transportation, mixing, sifting, dosing, sowing, and packaging are accompanied by repeated mechanical impacts of working elements and components of the appropriate equipment on loose material particles. As a result of excessive external dynamic and static loads, internal stress damage in the form of various types of deformation occurs within the particles of the bulk material.

**Figure 1:**
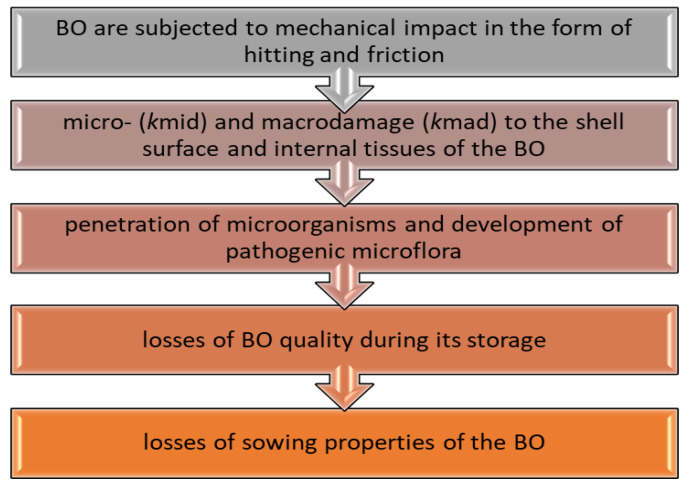
Changes in the quality of the BO considering their damage.

Minimization of internal damage is especially relevant for seeds of crops/biological objects (BO), where the damage level affects the realization of the crop’s reproductive potential. The current low coefficient of realization of the reproductive potential of corn seeds is 38–44%, which leads to inefficient use of resources, economic losses, technological depletion, and chemical contamination of soils [3]. The analysis of studies [4,5,6,7] highlights several significant consequences of mechanical damage to crop seeds. It was found that microdamage levels in grains during post-harvest handling range from 35% to 40%, with up to 23% of this damage attributed to grain cleaning and drying equipment. Damaged grain has nearly a 2-fold increase in respiration intensity, and its susceptibility to microbial contamination is 10–12 times higher. This damage can lead to a decrease in germination by up to 69%, plant survival by 10–15%, and plant yield by up to 28%. The study of causes and consequences of damage and the creation of appropriate methodology will allow us to predict and develop effective scientific and technical means to minimize the damage level and restore the BO biopotential. The typical loss of quality indicators of the BO, using agricultural crop seeds as an example, occurs according to the following algorithm (Figure 1).

The analysis of well-known methods for determining the mechanical properties of loose material particles showed difficulties in their use for the BO, which can be explained by the particularity of their structure [8]. The classical approach to the study of BO damage is based on the identification of macro- (external) and micro- (internal) damage by checking the strength condition: comparison of external contact forces and internal stresses [9,10]. When determining the BO strength, their elastic properties are significant, i.e., the material’s ability to return to its original shape [11]. The mechanical properties of the BO form a reaction to an external load, which is a combination of elastic, plastic, and ductile behavior. To identify these properties, precise laboratory testing is necessary [6,12]. The process of deformation and BO damage development depend on the static and dynamic nature of the external load applied by the working parts of technological equipment. Impacts during dynamic loading increase the risk of both internal and external damage. Studies of dynamics [13,14], which assess the strength properties of the BO, are less common and limited in practical results. This is due to the lack of a theoretical basis, the difficulty of developing universal methods, and the lack of necessary measuring devices. In order to determine the properties of the BO with the dynamic nature of loading, it becomes necessary to develop individual techniques and standards.

The considered BO are loose materials of biological origin and represent a group of particles that is characterized by significant deformation and dependence of their mechanical properties on moisture content [15,16,17,18]. Most of the obtained mathematical models of dynamics [19,20], storage [15], segregation and sifting [21], and cleaning of blocked holes [22,23] require accurate data on the strength properties of the BO. The following parameters [8,24,25,26] are used in assessing the BO strength: proportionality limit, elastic limit, biological yield strength, and biological strength limit. Therefore, in order to obtain reliable data, it is necessary to conduct preliminary sampling of the BO to ensure sufficient repeatability of the experiments.

The current identification of the BO damage is based on visual methods and the use of special equipment (microscopy, tomography, radiography, etc.) [7,27]. Visual methods allow to determine only the parameters of macroscopic damage placed on the BO surface in the form of dents, cracks, chipped parts, etc. The use of specialized equipment involves both chemical and physical methods of identification. The most promising are non-contact methods [28,29] and non-destructive techniques, namely machine vision, spectroscopy, hyperspectral imaging, soft X-ray imaging and thermal imaging. A common method of studying internal microdamage is radiography, which performs non-destructive analysis using complex X-rays [29,30,31]. The combination of non-contact methods, Acoustic emission (AE) and 4D X-ray computed tomography, for monitoring crack propagation in rocks has proven to be a successful tool for micro-level studies [32]. The use of such equipment is accompanied by difficulties: limitations in the objects’ size, calibration procedures, existing errors of each measuring element, and the complexity of experiments. A positive feature of non-destructive testing is the possibility of the subsequent use of the object for other studies; for example, determination of reproductive potential of the BO. However, the processing of the obtained images and numerical analysis of the internal damage parameters of the bodies causes a significant difficulty.

One of the image processing methods involved the use of statistical analysis to determine the porosity of the layer of vibrated fluidized loose material [33]. The image processing consisted of analyzing the areas of pores and grains and the probability of their placement. The essence of the Monte Carlo method consists of the statistical modeling of processes using a probabilistic model of an object in order to evaluate the studied integral characteristics based on the law of large numbers. Statistical modeling is widely used to solve problems [34,35,36] such as numerical integration, calculations in queuing systems, calculations of product quality and reliability, calculations of the passage of neutrons and other particles through matter, message transmission in the presence of interference, problems of game theory, problems of rarefied gas dynamics, problems of discrete optimization, and problems of financial mathematics.

The analysis of the conducted studies indicates the need to ensure adequate sampling, as well as the prospects of a non-contact method for determining the BO—using tomography followed by statistical image analysis and identification of the damage level. Compared to existing methods, this approach provides the necessary accuracy (suitable for practical applications and scientific studies) for determining the BO damage indicators, a shorter process duration, and the possibility of using stable, standard research equipment.

## 2. Materials and Methods

The research methodology involves preparing BO samples, subjecting them to experimental loading using a Zwick/Roell Z2.5 machine (Ulm, Germany) and an impact test stand with varied force parameters, performing tomography to assess internal damage, analyzing tomographic images statistically, and establishing relationships between microdamage levels and external load parameters.

### 2.1. Sampling

**Figure 2:**
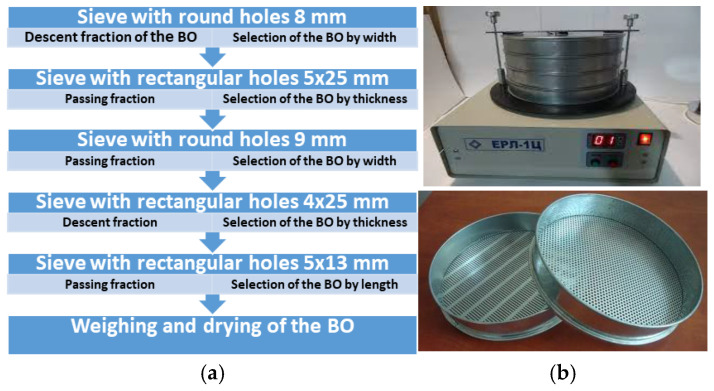
Selection of test samples by sifting: (**a**) scheme of laboratory sieves; (**b**) general view of laboratory sifter ERL-1C and sieves.

The studies were conducted on a BO of plant origin—seeds of corn hybrid P9074, which were gifted by Agricola-Lublin Ltd. (Ciecierzyn, Poland) and are currently one of the most popular crops in the world (the global corn area amounts to 208 M ha [37]). Morphological and agronomic properties of corn seeds P9074 are as follows: year of production—2023; purpose—grain production; seed type—toothed; potential yield—110 c/ha; maturity group—medium early; FAO—330; number of rows of seeds per ear—14–16 pcs. Corn BO is characterized by a complex morphological and anatomical structure, which directly determines their physical and mechanical properties. The following properties affect the mechanical (strength) characteristics of the BO and are influenced by the following properties [38,39]: size, shape, density, chemical composition, moisture, gluten and protein content, and vitreousness. These properties of the BO are determined mainly by the structural state of the main nutrient—endosperm, which for corn makes up to 86% of the particle volume [40,41]. The structure of the corn BO particle is characterized by a complex multi-layered structure and irregular outlines. This is influenced by the following factors [42,43,44]: biological (binding to a variety/hybrid, location on a swing, etc.), natural and climatic (soil fertility, suitable climatic conditions), and technological (quality of technological operations). The outer part of the BO particle is a multilayered membrane (epidermis, aleurone layer, etc.), which is responsible for protecting the embryo and endosperm. The sublayers of the membrane are formed by densely packed and relatively thick cellular elements [44]. The shell contains waxy compounds that make it relatively impermeable to water. This feature of the structure makes it possible to ensure long-term storage of corn BO without the risk of biological activation (germination). The main structural part of the BO is the endosperm, which contains protein and starch. These elements serve as the nutrient medium for the development of the BO during germination [44]. Changes in the shape and size of the BO samples during studies significantly affect the extent of damage, which can reduce the adequacy of measurements. Therefore, for sampling, preliminary sifting of corn BO particles on sieves was carried out (Figure 2), which made it possible to select a material with homogeneous properties.

**Figure 3:**
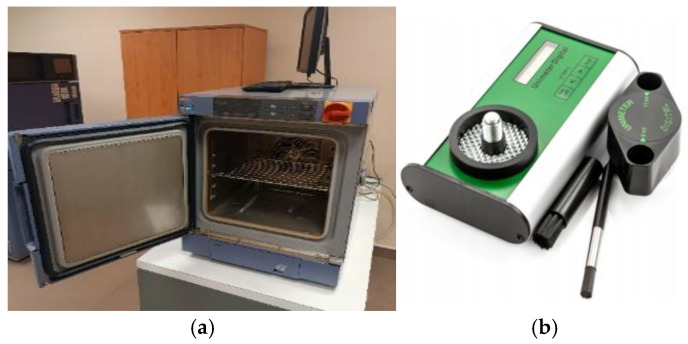
Sampling of the BO with the corresponding moisture content: (**a**) humidity chamber SH-661; (**b**) moisture meter UNIMETER 525525004.

For study reliability, moisture parameters of 14–15% are adopted, which meet the international quality standards DSTU 4138-2002, ISTA [45,46], and the requirements of classical production processes [47]. To obtain the required moisture content of the BO, we dried them in a humidity chamber SH-661 and checked them using a UNIMETER moisture meter 525525004 (Gloucester, UK, Figure 3).

Subsequently, a sample of BO was obtained, and its particle parameters were evaluated as follows: (1) geometric dimensions were measured using the custom-developed ImgToVal system [48]; (2) density was calculated through precision weighing (HE-500 scales, Scale Computing, Indianapolis, IN, USA) and volume measurement; (3) moisture content was determined using a UNIMETER 525525004 moisture meter; and (4) structural element parameters were examined through microscopy (OPTA-TECH x2000 microscope, Warszawa, Poland).

This made it possible to obtain homogeneous test samples of the BO with similar properties, which ensured the required accuracy (adequacy) of the research.

### 2.2. External Loading of the BO

The BO is affected by the influence of working elements and parts of technological equipment, which is mainly characterized by the static and dynamic nature of the load.

**Figure 4:**
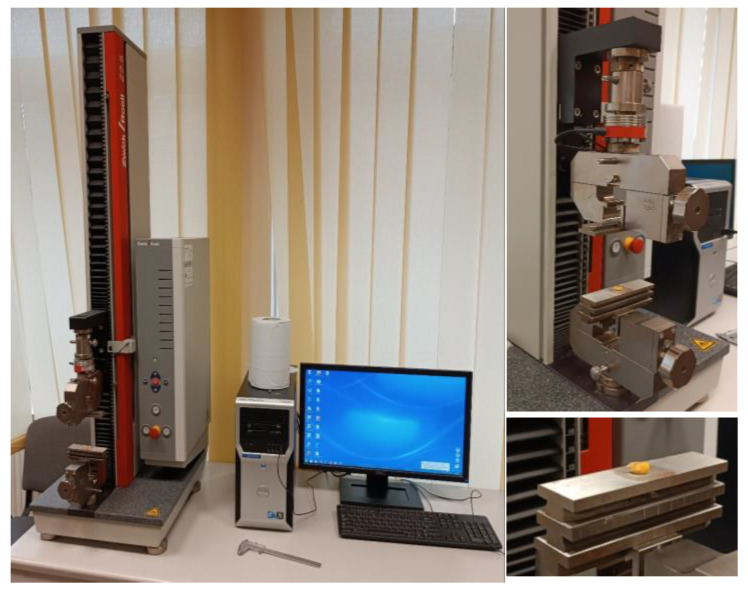
External static loading machine Zwick/Roell Z2.5.

The predominant approach to static loading is to measure the displacement of the working platform as a function of the load [8,49]. The studies are carried out under a low strain-rate and focus on the compression strain of the samples. To identify the parameters of the external static load, a typical Zwick/Roell Z2.5 stand was used (Figure 4, Table 1).

Static loading of the BO was conducted using the Zwick/Roell Z2.5 machine (Figure 4), with precise control and recording of both force and direction. This test bench is designed for automatic data processing in the testXpert II V3.3 program.

**Figure 5:**
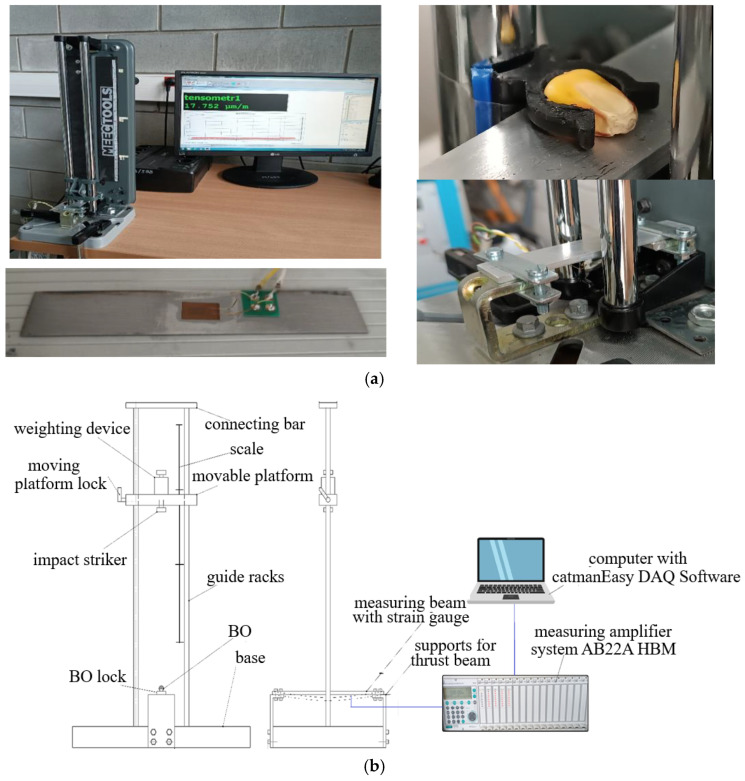
Developed laboratory equipment for external dynamic loading of the BO: (**a**) general view; (**b**) scheme.

Dynamic loading of the BO is accompanied by a rapidly changing nature of phenomena, the measurement of which is difficult when using standard measuring equipment. The complexity is represented by the small overall dimensions of the BO, their heterogeneous structure, and irregular geometric shape. Currently, the most common solution for implementing dynamic loading of the BO is the free-fall method [50,51,52]. The popularity of this approach is due to its simple design, the ability to regulate key measurement parameters, and its adaptability to the BO. Most studies on BO strength under dynamic loading have been conducted on devices that can be classified as follows [53,54]: (1) impact on the freely supported BO or (2) impact on the BO with support. A slightly different approach is presented in [13], where the use of an impactor in the form of a spring launcher is justified. The peculiarity of the developed test bench (Figure 5) was the possibility of regulating the weight and height of free-fall of the striker, the BO fixation angle, which made it possible to vary the impact velocity, and the magnitude and direction of the external load. Strain gauge measurements made it possible to numerically identify the force values during the interaction of the working element (striker or platform) of the stand with the BO, which corresponds to widely accepted approaches [55,56]. In the developed test bench used high-sensitivity strain gauges of the TF-5/350.

The signal processing from the strain gauge was carried out using the HBM MGCplus AB22A measuring amplifier system (Figure 5), with the results displayed in the Catman Easy 2.1 DAQ Software (Hottinger Baldwin Messtechnik, Darmstadt, Germany).

To ensure the required accuracy of the research, a preliminary study was conducted on a thrust steel platform with various elastic properties—thicknesses of 1, 2, and 3 mm. Further research was conducted on the 2 mm thick platform. To ensure the accuracy of the research, a special gripper (Figure 5) was manufactured (3D printing). The original test bench (Figure 5) made it possible to investigate the impact nature of the load on the BO.

**Figure 6:**
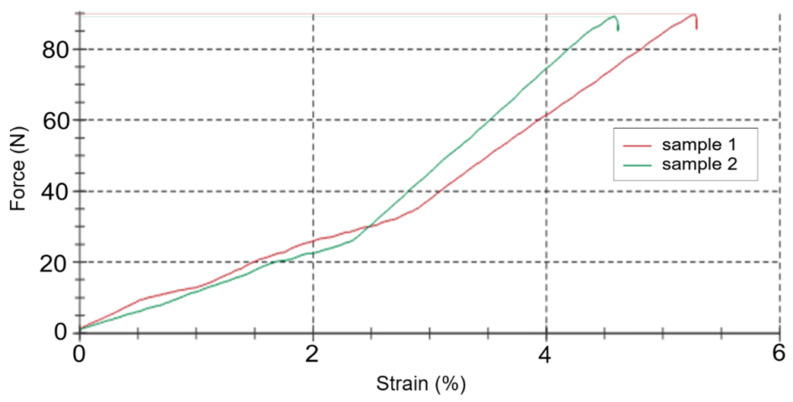
Example of measuring the loading force of the BO under static nature (Zwick/Roell Z2.5 machine, vertical arrangement of the BO).

**Figure 7:**
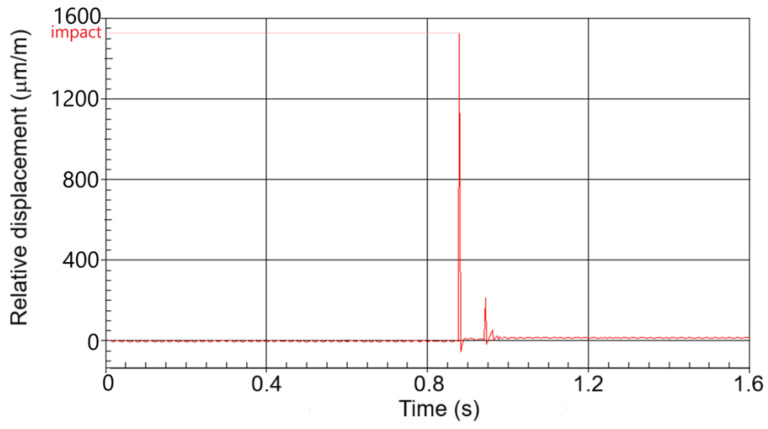
Example of measuring the load force on the developed impact test bench.

The measurement process on the developed stand (Figure 5) proceeds as follows: The installation was previously calibrated by loading the beam with the known standard weights, m_g_ = 10, 50, 100, 200, and 500 g. This made it possible to obtain a calibration curve of the beam displacement versus the applied force (F = m_g_g). Next, the BO, calibrated by size (shape), was installed in a clamp (Figure 5) in the appropriate vertical or horizontal position. According to the measurement plan, the required weight was further set—0, 45, 185 or 325 g. The platform was fixed with the help of a blocker pos. 8 at the required height (scale). In catmanEasy DAQ Software, before each measurement, zeroing was carried out and the platform was started. The maximum value of the displacement of the platform was recorded and the force magnitude was determined by the calibration curve. Examples of the obtained patterns of the magnitude of external loading from time are shown in Figure 6 (for static nature) and Figure 7 (for dynamic).

An important stage of the measurements was determining the critical load (F_kr_) of the BO, at which full deformation occurred. Complete deformation was characterized by the destruction of a particle with external signs of damage. The Zwick/Roell Z2.5 machine, with a loading platform speed of 5 mm/min, was used for this purpose. This made it possible to determine the critical force at which deformation of the BO sample occurred. Further, three loading options were used to simulate the BO damage: 0.3 F_kr_; 0.45 F_kr_; 0.6 F_kr_; 0.75F_kr_; 0.9 F_kr_. For static loading on the Zwick/Roell Z2.5 machine, this option is available in Catman Easy DAQ software. For the developed test bench with dynamic loading, the variation in the force magnitude (0.3–0.9 F_kr_) occurred by changing the drop height and the weight of the weighting device (Figure 5).

**Figure 8:**
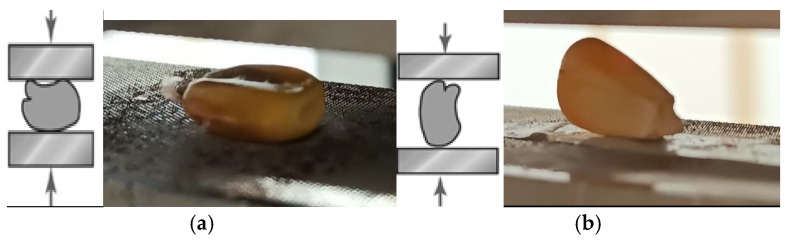
Options of the BO arrangement at external loading: (**a**) horizontal; (**b**) vertical.

The research tasks also included studying the position of the BO relative to the striker in two installations. Two variants of the arrangement were used (Figure 8): sideways relative to the width and sideways relative to the thickness. These positions are typical for contacts with the working elements of technological equipment [57]. The stable position of the grain was ensured by the double-sided adhesive tape.

### 2.3. Tomography of Damaged BO

**Figure 9:**
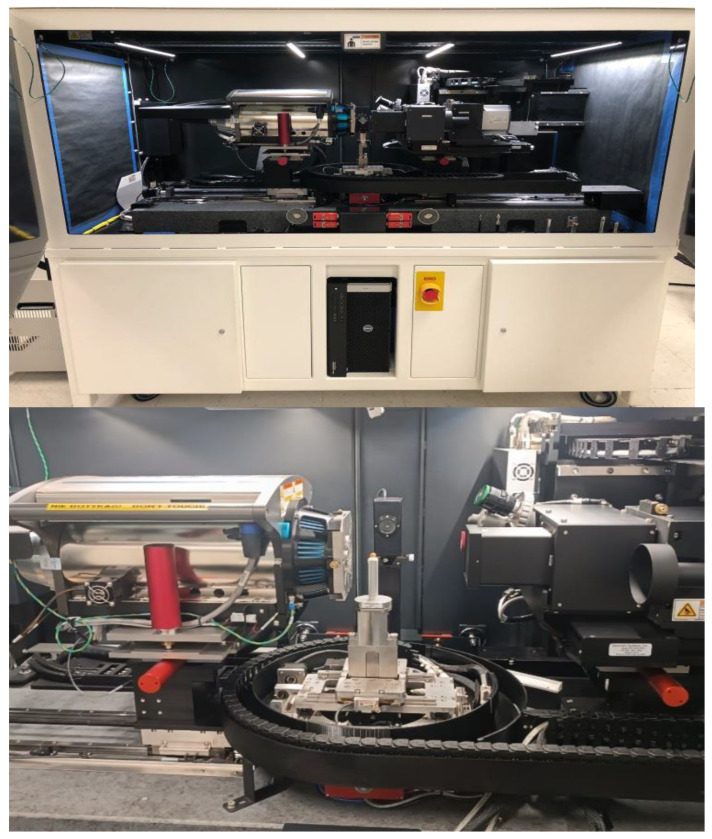

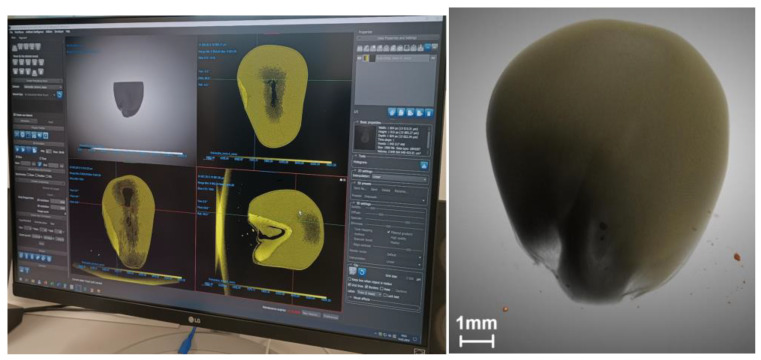
Tomography of the BO samples on the Zeiss Xradia 510 Versa 3D tomograph.

The next stage was tomography of previously damaged samples of the BO. For this purpose, the Zeiss Xradia 510 Versa 3D tomograph (Jena, Germany) was used (Table 2, Figure 9), which allows us to obtain images of the inner part of the BO without contact with the required quality.

The use of the ZEISS Xradia 510 Versa tomograph is justified by the high resolution 3D imaging (the resolution of the system achieves < 0.7 μm true spatial resolution with minimum achievable voxel size < 70 nm of samples) without destroying the BO. Xradia 510 allows us to obtain submicron images.

The following ZEISS Xradia 510 Versa settings were used for the work: 4× lens that can work with samples from 2 to 50 mm; maximum 3D FOV (WFM)—6(10) mm; voxel size—0.7–3 µm. The average tomography time for the BO with the given parameters was 3.4 h.

The result of this stage was a series of 2D images (10 evenly spaced horizontal and 10 vertical sections of the BW) and a 3D image. For better visualization of the internal damage (cracks), inversion of the BO color was used.

### 2.4. Data Processing Using the Monte Carlo Method

To process the obtained 2D tomographic images of the previously damaged BO, the method of static tests or the Monte Carlo method in the context of geometry identification on the plane was used. Such a method is based on checking overhead points (grids) for a geometric object to be identified [59,60,61]. With a significant number of superimposed *N* points, the part of the points that hit into the damage (crack) is equal to the ratio of this figure’s area to the area of the BO.

The following data processing algorithm is used:

1. Build a grid on the 2D image of the BO with a uniform distribution and generate the coordinates of the point (*x*, *y*).
2. Check if the point (*x*, *y*) belongs to the shape (damage) that we are defining. If there is no hit, i.e., at least one of the system inequalities is not satisfied, then go to step 1 and generate the coordinates of the new point. If we have a hit, we fix it. The value of the hit counter increase the number of hits and go to point 1 again.

Note that hitting a point exactly on the damage boundary causes an intermediate situation.

Points 1 and 2 must be repeated a significant number of *N* times in the cycle. The accuracy of calculations depends on this. After performing repetitions, we determine the area of microdamage.

The accuracy of the method is determined by the error, the value of which is directly proportional to the number of accepted points *N*.

For a comprehensive assessment of internal damage level in the BO, the microdamage coefficient Kmd was used. This coefficient reflects the number and size of internal cracks relative to the surface area of the BO.

**Figure 10:**
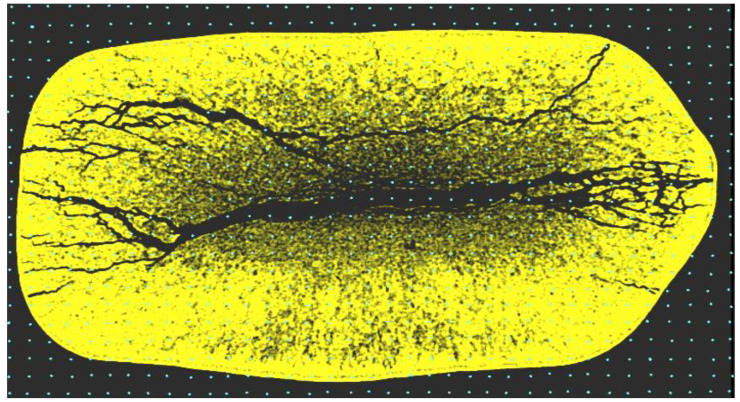
Image of the damaged BO (*N* = 3400).

The crack propagation inside the BO is complex and the intensity of placement differs for each resulting cross-section of the BO. The identification of the microdamage coefficient was performed at each cross-section of the BO according to the Monte Carlo statistical testing method, using the following expression (Figure 10):

(1) $$K_{mdi}=1-\frac{\sum_{i=1}^{N}\xi_{i}}{N},$$

where *N*—points distributed throughout the object (with *N* = 3400 accepted for one of the BO); $\xi_{i}$—random variable; *i*—point number.

When a point falls within a damage (crack) of the BO, $\xi_{i}$ = 0, and if it is on the boundary, $\xi_{i}$ = 0.5. When a point falls within the image of a solid material, $\xi_{i}$ = 1.

The overall microdamage coefficient of the BO was calculated as the arithmetic mean:

*K_md_* = (*K_md_*_1_ + *K_md_*_2_ + … +*K_mdj_*)/20, (2)

where *K_md_*_1…*j*_—microdamage coefficients of horizontal and vertical cross-sections of the BO.

The actual value of the microdamage coefficient was determined using the following expression:

(3) $$K_{fmd}=\frac{V_{dam}}{V_{BO}},$$

where *V_dam_* is the volume of microdamage in the BO and *V_BO_* is the volume of the BO.

The BO volume, VBO, was determined by the volume of liquid poured into a measuring container with 100 pieces of the BO placed inside. The volume of microdamage (*V_dam_*) was identified by determining the damage area (cracks).

The accuracy of the measurement of *K_md_* was assessed based on the magnitude of the confidence interval under normal distribution of the error:

(4) $$\left|\frac{\sum_{i=1}^{N}\xi_{i}}{N}-K_{md}=K_{fmd}\right|<x_{\beta }\sqrt{\frac{D_{\xi }}{N}},$$

(5) $$\mathrm{at}\;D_{\xi }=\frac{1}{N}\sum_{i=1}^{N}{\left(\xi_{i}\right)}^{2}-{\left(\frac{1}{N}\sum_{i=1}^{N}\xi_{i}\right)}^{2}.$$

where $x_{\beta }$—quantile of the normal distribution (thus, at a confidence level *β* = 0.95, $x_{\beta }$ = 1.96); $D_{\xi }$—variance of the estimate.

## 3. Results and Discussion

As a result of the preliminary calibration of the BO (according to the methodology in Section 2.1), samples with the following identical properties were obtained (Table 3): thickness, width, length, density, moisture content. This allowed for the minimization of the impact of the BO properties on the final result.

**Figure 11:**
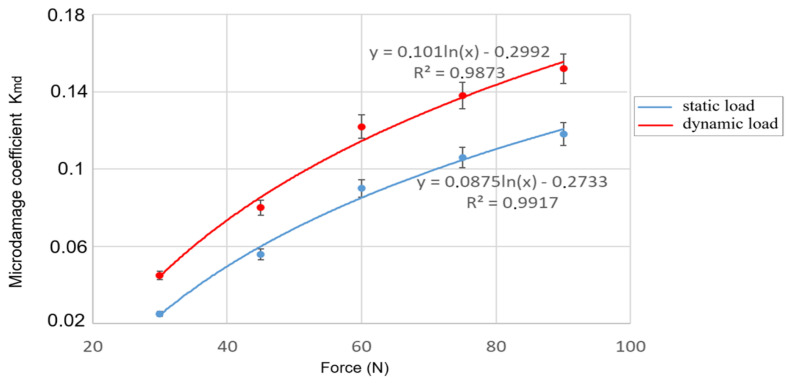
Dependencies of the BO microdamage level on external static (blue) and dynamic (red) loads with the vertical arrangement of the BO.

**Figure 12:**
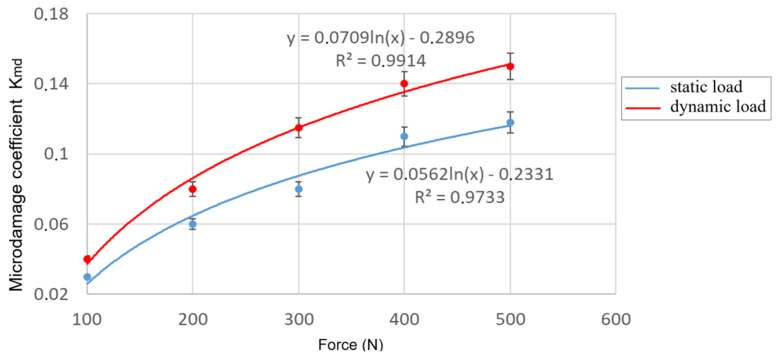
Dependencies of the BO microdamage level on external static (blue) and dynamic (red) loads with the horizontal arrangement of the BO.

The use of the proposed methodology, specifically the artificial loading of the BO, their tomography, and image processing using the Monte Carlo method, allowed for the establishment of dependencies of the BO microdamage coefficient on the parameters of external load (Figure 11 and Figure 12).

The obtained data on microdamage of corn seeds are partially consistent with the data in [62]. In contrast to a number of existing methods [63,64], where the authors use an external load in the form of tied kinematic parameters (rotation frequency of the machine’s working element or its velocity of movement), we propose to use the force value, with different types of its application to the BO. By determining such a force, this approach significantly enhances the practical relevance of the methodology and its results.

The analysis of dependencies (Figure 11 and Figure 12) revealed a significant influence of the selected factors (magnitude and nature of external load, position of the BO) on the intensity of microcrack formation. Small external forces are needed for the damage to occur in the vertical position of the BO (Figure 11), and the horizontal position required greater external forces (Figure 12). It was established that the highest damage and intensity of cracks formation in the BO occurs under impact loading.

Increasing the magnitude of the external load increases the damage level by 5.5–6 times under static conditions and by 3–3.3 times under dynamic conditions.

Knowing the type and magnitude of external forces, by using our methodology we can predict the probable level of the BO damage and exclude it.

Determining the magnitude of external forces from the action of working bodies (blades, walls, scrapers, screws, etc.) is not difficult today. Combining these data with the proposed methodology (and in the case of corn seeds, with the established dependencies), we will obtain the amount of probable damage. Further improvement of technological equipment may consist in reducing the velocity of movement of loose material particles or using additional elastic surfaces on the working bodies.

Further development of this work involves studying the impact of the BO properties (moisture content, density, and the ratio of structural elements) on the level and localization of their damage, as noted in [63,65]. Additionally, the proposed methodology may enable the determination of the effects of multiple loads of the BO, as noted in [6].

Further research on BO damage, taking into account the presented methodology and data, may consist of a point cloud and its integration into an FE model. The results obtained from the FE model can be compared with the experimental data obtained from corn seeds. This will allow us to investigate the influence of the structures of a BO on crack propagation and micro-damage levels without lengthy experiments.

## 4. Conclusions

To study the BO damage levels, a methodology has been created, which is based on the sequential implementation of the following stages: preparation of the experimental BO samples; experimental loading of the BO samples on a Zwick/Roell Z2.5 static machine and a developed impact stand with varying the magnitude and direction of the external force; tomography of the BO samples subjected to the specified loading; statistical analysis of tomographic images based on the Monte Carlo method; data processing and determination of dependencies between the BO micro-damage level and the parameters of the external load.

The use of standard equipment alongside the developed impact stand and the use of a static data processing method helped reduce the test time and improve measurement accuracy.

The variable parameters in the research are as follows: static and dynamic type of external load, variation in its value, vertical and horizontal placement of the BO, which allow to maximize the use of results for practical and scientific purposes.

As a result of testing the proposed methodology, dependencies of the microdamage coefficient of corn seed BO on its location and parameters of external loading were obtained.

The research results, presented as a methodology and obtained data, are valuable for the following areas: improving the quality of loose materials by reducing damage, enhancing technological equipment, conducting studies on predicting potential BO damage, and optimizing the parameters of technological processes for handling loose materials based on an additional criterion of damage level.

## Figures and Tables

**Table 1:** Technical characteristics of the Zwick/Roell 2.5 machine.

Technical Specifications	Value
Test load maximum, kN	2.5
Drive travel resolution, μm	0.0277
Initial force, N	1
Speed of measurement, mm/min	5

**Table 2:** Equipment specifications the ZEISS Xradia 510 Versa.

Paratemer	Value
X-ray source	30–160 kV, 10 W maximum power, 12 filters for energy selection
X-ray detector	Dual-stage detector system with 2k × 2k pixel. The detector turret of multiple objectives (0.4×, 4×, 20×) at different magnifications with optimized scintillators for highest contrast
Spatial resolution	<0.7 µm true spatial resolution and below 70 nm voxel size
Reconstruction	XMReconstructor
Analysis software	Dragonfly Pro [58]
Radiation safety	<1 µSv/h at 10 cm from the instrument surface

**Table 3:** Properties of the BO particle samples.

Properties	Unit of Measurement	Value	
		Min	Max
Density	kg/m^3^	618.13	622.35
Length	mm	12.09	13.11
Width	mm	8.02	9.07
Thickness	mm	4.1	5.05
Moisture content	%	14.92	15.11

## Data Availability

Data are available upon reasonable request to the authors.
